# Calibrating facial morphs for use as stimuli in biological studies of social perception

**DOI:** 10.1038/s41598-018-24911-0

**Published:** 2018-04-27

**Authors:** Sonja Windhager, Fred L. Bookstein, Hanna Mueller, Elke Zunner, Sylvia Kirchengast, Katrin Schaefer

**Affiliations:** 1 Department of Theoretical Biology, University of Vienna, Vienna, Austria; 2 Department of Evolutionary Anthropology, University of Vienna, Vienna, Austria; 3 Department of Statistics, University of Washington, Seattle, USA

## Abstract

Studies of human social perception become more persuasive when the behavior of raters can be separated from the variability of the stimuli they are rating. We prototype such a rigorous analysis for a set of five social ratings of faces varying by body fat percentage (BFP). 274 raters of both sexes in three age groups (adolescent, young adult, senior) rated five morphs of the same averaged facial image warped to the positions of 72 landmarks and semilandmarks predicted by linear regression on BFP at five different levels (the average, ±2 SD, ±5 SD). Each subject rated all five morphs for maturity, dominance, masculinity, attractiveness, and health. The patterns of dependence of ratings on the BFP calibration differ for the different ratings, but not substantially across the six groups of raters. This has implications for theories of social perception, specifically, the relevance of individual rater scale anchoring. The method is also highly relevant for other studies on how biological facial variation affects ratings.

## Introduction

This paper proposes an approach for calibrating facial stimuli that allows facial image input to be formalized by constraining variation solely to the biological or appearance variable of interest. The variable here is body fat percentage [BFP] (usually highly correlated with body mass index [BMI], the more familiar but less physiologically accurate quantification^1^ of body fat). This formalization enables systematically creating virtual faces corresponding to any shape regression of interest that can then be rated for any selected property. “To calibrate is to use empirical data and prior knowledge for determining how to predict unknown quantitative information Y from available measurements X via some mathematical transfer function.” (Martens and Naes^2^, page 2) This is a standard definition out of engineering statistics. In our application, Y is the expected picture at some given BFP, the measurements X are the sample’s BFP’s plus the facial shape coordinates, and the transfer function is the ordinary shape regression. The specification of that mathematical model is actually the first step in calibration; the second is the acquisition of the instrument data X (here, our BFP’s) and the data Y to be estimated (the shape coordinates), and the third is often some application of covariance analysis such as linear regression or partial least squares^2^.

The idea of calibrating stimuli was introduced about 150 years ago to bring the certainty of machines into psychology. Four German scientists trained in physiology initially applied the experimental method to the mind, the subject matter of the new psychology: Hermann von Helmholtz, Ernst Weber, Gustav Theodor Fechner, and Wilhelm Wundt (cf.^3^). Even as far back as the 19th century, the curves of individual responses to systematic variation of stimuli under the experimenter’s control were expected to greatly enhance our understanding of the human nervous system. Note that the “data” here were whole curves, not single points.

Contemporary face research embraces certain themes of this old psychometric work, in particular the intentional bridging of biology with psychology in order to dig deeper into the physical brain by careful experimental study of subjects’ subjective reports. The currently dominant methodologies in this domain find associations of specific facial features with specific ratings. Until now, however, there has been no straightforward way to report consistent patterns among these numerous associations. This gap partially reflects an incomplete stimulus calibration step. Social perception studies of facial adiposity may serve as illustration: (1) Sometimes stimuli are not quantified at all when the correlation coefficient between BMI or BFP values and facial ratings is the statistical measure^4,5^. (2) Whether for stimulus production or for the visualization of shape-trait associations, the calibrated (predicted) images are preferable over any sort of tail-averaged images (such as^6–8^). The possibility for calibration legitimately extends to any other kind of exogenous measure (gathered independently of the photograph), like body height or sociosexual orientation, conventionally also depicted as tail-averages (e.g.,^9^). A case in point is the typical response of raters to facial images near the sample average (Appendix 1). The present contribution fills this gap in the design of rating studies by controlling for characteristics of the stimulus and the rater at the same time. (Readers familiar with test theory may recognize this strategy as analogous to a combined Rasch scaling of ability and item difficulty in some approaches to school testing.^10^) To achieve the necessary control we produce images that vary *solely* in the facial shape consequences of the variable of interest, in our case BFP, rather than the innumerable other individual differences that usually blur the stimulus variable.

The topic of BFP’s facial correlates is a suitable choice for demonstrating the method because facial correlates of BMI were the most integrated pattern^11^ and at the same time contribute to current research into the social consequences of obesity or its opposite, which we refer to here as “leanness.” We modified the image unwarping and averaging previously explained in Windhager *et al*.^12^ to yield a series of five facial configurations of female adolescents calibrated by BFP. These were presented in a rating experiment in order to determine whether the larger, rounded mid- and lower face with increased BFP elicits associations with (i) babies’ chubby cheeks^13,14^, or rather with (ii) masculinity and dominance due to the relative jaw prominence^15^. Evolutionary aesthetics^16^ posits that health and attractiveness ratings should be highest for moderate amounts of body fat. We did not predict systematic differences between male and female raters or among different age groups (adolescents, young and older adults).

## Materials and Methods

### Raters

In 2013 and 2014, 274 men and women completed the rating study during daytime hours on working days in offices and classrooms in Vienna. One additional rater self-reported a nonbinary gender. That subject was omitted from our data. We classified the participants into one of three age groups. The *Adolescents* group included 58 males and 48 females aged 14 to 18 years. There were 115 *Younger Adults* (mainly students), 54 men and 61 women aged from 18 to 35, and, finally, 53 *Older Adults:* 27 men and 26 women aged from 37 to 76 with an average age of 54. Further descriptive statistics are given in Table 1. This yielded six rater groups. Each participant was informed about the rating procedure and the right to withdraw from the study at any time. Data collected were recorded against an anonymous study ID, and the protocols were in accordance with the Declaration of Helsinki.

**Table 1:** Descriptive statistics of age for the six groups of raters.

Group	Age (years)							
	Men				Women			
	*M*	*SD*	*Range*	*n*	*M*	*SD*	*Range*	*n*
Adolescents	15.60	0.92	14–18^a^	58	15.98	0.70	14–17	48
Younger Adults	25.65	4.46	19–35	54	23.97	4.75	18–34	61
Older Adults	56.37	9.72	38–76	27	52.81	7.37	37–68	26

*Note*: ^a^A single 18-year-old male participant still attended secondary school and was assigned to the adolescent group.

### Stimuli

**Figure 1:**
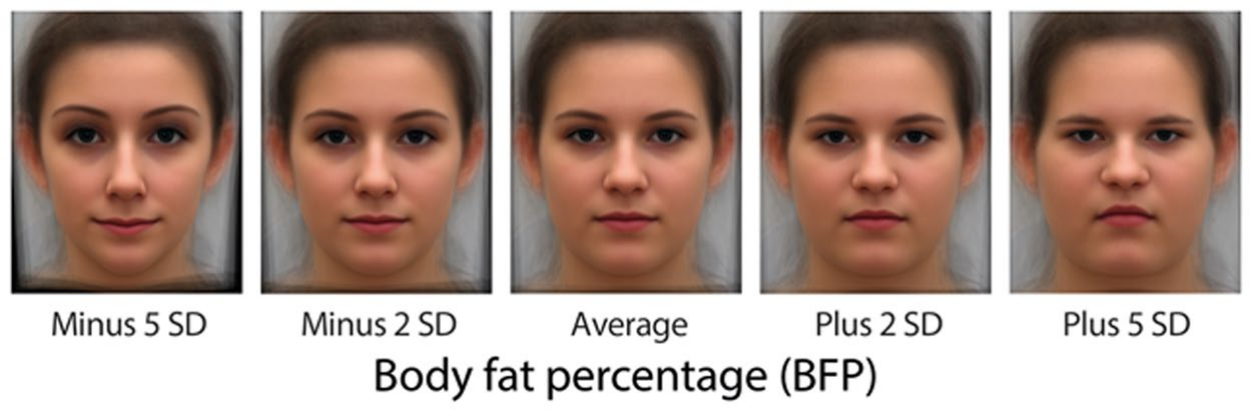
The face stimulus set (calibration sample) varied solely in the amounts of body fat percentage (BFP) associated with facial shape. From the geometric morphometric shape regression upon body fat percentage in female adolescents originally published in Windhager *et al*.^12^, landmark coordinates of five target configurations were computed for the sample average as well as the average plus and minus 2 and 5 standard deviations of BFP. The original photographs of the adolescents were then unwarped to these target configurations and averaged. Apparent changes along this progression in small features (e.g., eyebrow arching) are epiphenomena of the underlying regression of shape on BFP, a pattern showing large-scale integration^11^. They are not experimentally controlled or otherwise manipulated features of the stimulus ordination per se.

The shape regression on which the stimulus set is based was originally presented in Windhager, Patocka, and Schaefer^12^. Twenty-two images were acquired by a Canon EOS 40D digital camera placed at subjects’ eye level 355 centimeters directly in front of their face in Frankfort horizontal orientation (top of earhole and bottom of orbit at the same height). The focal length was 200 mm. The female adolescents were instructed to gaze directly at the camera with a neutral (i.e., resting) facial expression. Images were reduced to 72 homologous points^12^ and the configurations converted to their Procrustes shape coordinates by the usual algorithm^17^. Each image was unwarped to the Procrustes average of these 22 configurations and the unwarped images then averaged in turn. In addition to these landmark configurations, we used conventional bioelectrical impedance analysis (via a Tanita TBF 105 body composition analyzer with bipolar foot-electrodes) to estimate body fat percentage (BFP) for each of the subjects^18^. These estimates ranged from 10.6% to 44.8% with a mean of 22.7% and a standard deviation of 8.4%. The 22 landmark configurations were regressed on BFP in the usual manner, shape coordinate by shape coordinate, and the predicted configurations computed for BFP scores differing from the mean by ±2 SD and ±5 SD produced in addition to the Procrustes mean. Finally, each photograph was unwarped to each of these five BFP-predicted shapes. Then these pictures were averaged for each target shape^19^. This procedure informs the morphs by the whole original sample, not just by tails of a BFP distribution. The resulting set of images, which we term the calibration sample, is laid out in Fig. 1.

Each of 274 raters was asked to rate each of these five morphs on each of five scales (in German): Maturity (child – adult), Dominance (submissive – dominant), Masculinity (feminine – masculine), Health (unwell appearance – healthy appearance), and Attractiveness (hardly at all attractive – very attractive). The sequence of morphs was randomized over raters. The order of the ratings themselves was first Health, followed by Masculinity, Attractiveness, Dominance, and Maturity, interspersed with six distraction scales, and the poles for Dominance exchanged from those in the figures to follow. Rating scales were presented simultaneously. All visual analogue scales ranged from 0 to 100 (numbers not visible to participants) and positions were marked using onscreen sliders or by directly indicating a point.

### Data display and graphical summary

**Figure 2:**
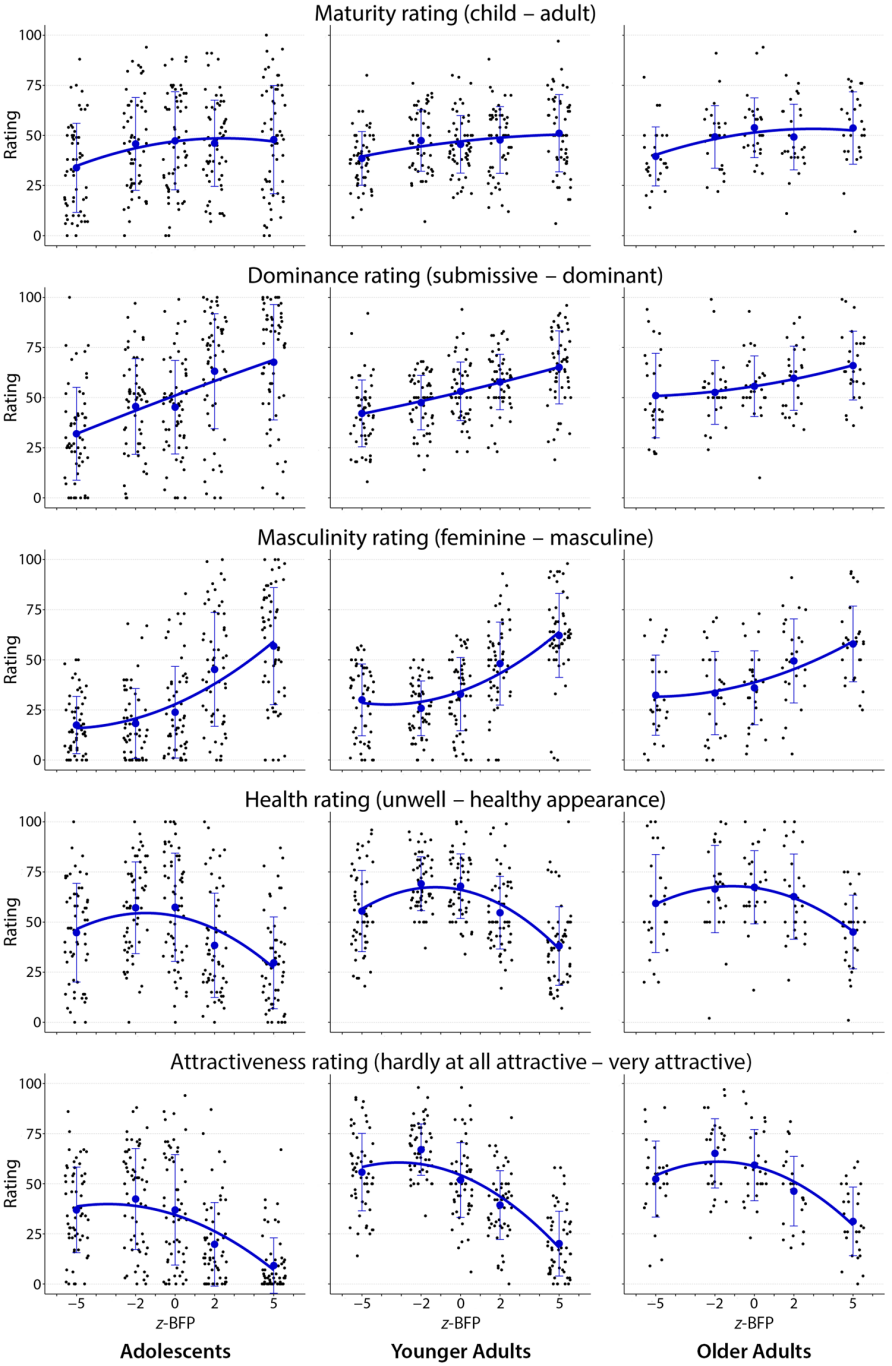
Male participants’ rating of the five female adolescent morphs. The *y*-axes depict the range of the continuous rating scales. The *x*-axis bears the *z*-scored body fat percentage (*z*-BFP) of the five facial shapes driving the morphing. All scatterplots depict mean ± one standard deviation rating per stimulus, overlaid by a least-squares quadratic fit. There is high consistency in curve shape across the three age groups, i.e. within a row, and distinctness across the five rating scales, i.e. down the rows.

**Figure 3:**
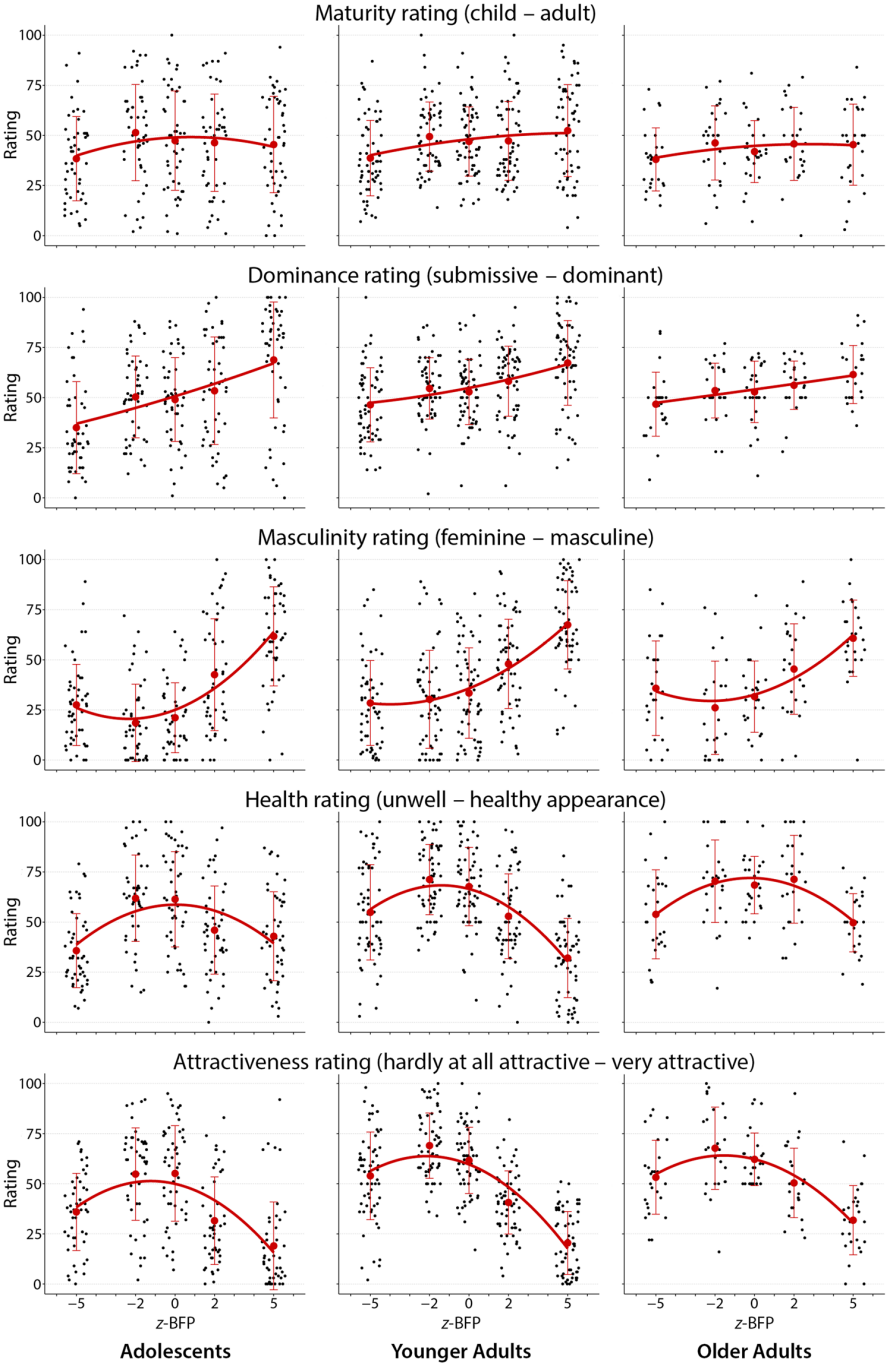
Female participants’ rating of the five female adolescent morphs. These fifteen scatterplots depict the original data of the female raters analogously to Fig. 2.

The above design yielded a complicated data structure: five levels of stimulus crossed by five different rating scales crossed by six different groups of raters. We adopt an approach familiar from contemporary data science: Explicit display of all the project’s data in a single diagram. Figs 2 and 3 together present the ratings of all 5 calibrated morphs by our 6 groups of raters on each of the 5 normalized rating scales. Each of the 30 panels (gender by age group by rating) is overlaid by a suggestive graphical summary, the quadratic regression of each panel’s data on BFP, which was the calibration parameter driving the morphing of the stimuli.

Within each panel, each dot is one scale score for one rater rating one stimulus on one characteristic. The dots are arranged in columns according to the stimulus (morphs corresponding to −5 SD, −2 SD, 0 SD, +2 SD, or +5 SD of BFP from the average). Within these columns, horizontal position corresponds only to the graphic trick known as “jittering,” the use of random variation to avoid pixel overlap. Each column is summarized by a conventional bar at ±1 SD of the rating, and each panel is summarized by the conventional least-squares quadratic fit.

### Statistical analyses

**Figure 4:**
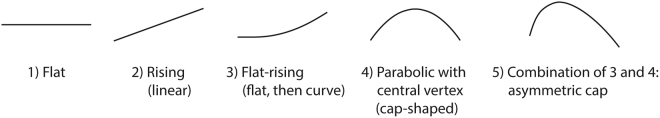
Selected curve prototypes. Calibrated stimuli allow fine-graded quantitative models of response patterns for the different rating scales. In our case, the individual ratings were best summarized by linear and quadratic regression fits. This figure highlights the different curve shapes resulting from the combination of our calibrated morphs upon BFP together with the request for five different ratings of each morph. While the absolute position (of the vertex) along the rating scale or the slope varies somewhat by rater sex and age group, the overall shape of the curve as depicted here is remarkably constant across the rows of Figs 2 and 3.

Linear and quadratic regressions were computed for each of the five rating scales for each of our six subject groups. The *R*^2^ values for these regressions in each panel are tabulated in Table 2, but they are not our main quantitative focus. Our emphasis is on the *shapes* of those quadratic regression fits. Out of the range of possible forms of these curves, we encounter five actual varieties in these data: (1) essentially flat trends, (2) linear rising/falling trends, (3) trends that are flat at one end but then curve upward/downward, (4) trends that are parabolic with vertex centered over our range of morphs (cups facing upward, or caps facing downward), and (5) combinations of (3) and (4), representing asymmetric cups or caps. See Fig. 4 for prototypes of these five.

**Table 2:** Coefficients of determination for linear and quadratic fits, ratings by BFP within age-sex groups.

Fraction of variance explained*						
Rating	Adolescents		Younger Adults		Older Adults	
	♂	♀	♂	♀	♂	♀
**Maturity**						
linear	0.0295	0.0040	0.0520	0.0368	0.0630	0.0144
quadratic	0.0424	0.0205	0.0551	0.0398	0.0814	0.0193
**Dominance**						
linear	0.1926	0.1526	0.2117	0.1189	0.0871	0.0975
quadratic	0.1927	0.1531	0.2121	0.1215	0.0920	0.0975
**Masculinity**						
linear	0.2780	0.2206	0.2812	0.2563	0.1834	0.1567
quadratic	0.3016	0.3254	0.3355	0.2985	0.2012	0.2390
**Attractiveness**						
linear	0.1816	0.0885	0.3488	0.2933	0.1617	0.1554
quadratic	0.2228	0.2421	0.4427	0.4621	0.2903	0.3250
**Health**						
linear	0.0611	0.0001	0.1069	0.1301	0.0439	0.0029
quadratic	0.1336	0.1323	0.2778	0.3037	0.1336	0.1815

^*^For linear regressions, this is *r*^2^; for multiple regressions, the conventional *R*^2^.

The absence of conventional statistical significance testing throughout this manuscript reflects the complex design of this study, which does not correspond to any conventional multivariate testing scheme. One of us (FLB) has written at length about the irrelevance of conventional statistical significance testing to attempts at knowledge discovery in the biological sciences^17^; this irrelevance extends to multidisciplinary projects partly grounded in biology as well. Formulated differently: while the null hypothesis of no differences over the 30 curves of this diagram is untenable and hence not worth “testing,” there is no well-formed alternative hypothesis or family of alternatives, and hence no valid way to convert any apparent pattern, whatever it may be, to a likelihood ratio. We further justify the absence of conventional *p*-values in Appendix 2.

We used Microsoft Excel (with Visual Basic Editor) for stimulus presentation, and the tps program series of F. James Rohlf^19^ for landmark digitization, Procrustes superimposition, sliding, regressing on BFP, and image unwarping and averaging. The curve prototypes were created in Adobe Photoshop CC 2017. Scatterplots, quadratic regressions, means, and standard deviations were realized in *R* 3.3.2^20^, relying on the packages *ggplot2*^21^ and *cowplot*^22^.

### Data availability

All data generated or analyzed during this study are included in this published article (and its Supplementary Information files).

## Results

### Distinct response curves for the five attributed traits, independent of rater age and sex

The main conclusion that follows from Figs 2 and 3, the enhanced display of the entire raw data base, can be summarized in two sentences. (I) For all five rating scales, the form of the quadratic regression of these normalized data upon our morphing parameter, body fat percentage, is the same for all six age-sex rater groups. (II) The form of those common response curves, however, varies considerably over the five rating scales: flat for the maturity rating as a function of morphing parameter, linear sloping upward for the dominance parameter, a combination of these two patterns for masculinity, cap-shaped for health, and a combination of flat-downward with the cap for attractiveness (for fractions of variance explained see Table 2). It is this paired main finding – the similarity of these trend curves across the columns together with their dissimilarity down the rows of Figs 2 and 3 – that contraindicates any envelope significance test, as explained above.

In a nutshell, there was no dependence of maturity ratings on BFP, a positive linear association with dominance attributions (the more the BFP, the more dominant), and curvilinear patterns for perceived masculinity, health, and attractiveness with moderate amounts of BFP being rated as more feminine, healthy, and attractive than extremely low or high amounts of BFP. While linear models are well suited for summarizing maturity and dominance attributions, quadratic regressions clearly outperform the linear ones when it comes to masculinity, health, and attractiveness ratings.

### Scrutinizing the response curve shapes

The most conspicuous aspect of this 6850-point composite display is its variation from row to row (three basic patterns plus two combinations). From top to bottom, these are the *flat* pattern (row 1), the *rising* pattern (row 2), a *flat-rising* mixture pattern (row 3), a *cap-shaped* pattern (row 4, with exceptions), and a *flat-falling* mixture pattern, row 5.

ROW 1, “child–adult,” *flat*. Regardless of rater group, the rating scale “child–adult” shows very little net dependence on the BFP morph parameter. The issue is not whether these curves do or do not differ “significantly” from end to end or from rater group to rater group—that is mainly a function of sample size, which is not evolutionarily meaningful—but whether their amplitude is large enough to be worth describing in the context of the shapes of the other four rating scales. By comparison with the descriptions to come, the top row of Figs 2 and 3 should be deemed a collection of curves that is simply uninformative: no interesting variation in their heights by morph parameter either within or between rater groups, and ratings spanning the full scale range throughout. Summarizing: for whatever reason, inducing the behavior of producing a rating on a scale labeled “child–adult”, whatever neural processing is involved in producing that behavior, seems unlikely to be yielding useful information about the evolutionary psychology of body fat.

ROW 2, “submissive–dominant,” *rising*. The uppermost set of panels contrasts strikingly with the next row down: The rating scale submissive–dominant clearly has a positive association with BFP. The quadratic fit curves in this row are all indistinguishable from their linear component alone (here, as throughout this paper, we refer not to statistical significance but to scientific meaning). The slopes of these lines are well approximated by their tenfold magnification, which is the difference of mean scale score between the extreme morphs at ±5 SD of BFP. Left to right, these differences are 36, 23, 16 scale points in male raters (Fig. 2), and 34, 21 and 15 points in female raters (Fig. 3). The slopes thus fall from left to right across the columns of the page. In other words, ratings by older raters show much less dependence on the morph parameter than ratings of the adolescent age peers.

ROW 3, “feminine–masculine,” *flat-rising*. A third pattern of dependence on the morph parameter is illustrated in the third row of panels, those for the rating scale feminine–masculine. This pattern appears as a blend of the first two: flat at the low-BFP end of the morph parameter scale, becoming steeper at the upper end. (In settling on this description we intentionally ignore the relatively weak evidence of non-monotonicity for the −5 morph stimulus as rated by the female peers and the older women.) Male adolescent raters and male young adults are particularly definitive about the femininity of the morphs of below-average BFP, never rating them above 57 on the rating scale (where highest masculinity is coded as 100). The other rater groups are less consistent but demonstrate comparable differences of mean rating across the 10-SD range of these morphs. Generally, morphs at average and below-average BFP are rated as markedly feminine, whereas above the average BFP, perceived masculinity increases with BFP.

ROW 4, “health,” *cap-shaped*. The average health ratings for the extreme morphs at ±5 SD of body fat percentage are comparable in all rating groups, and are substantially lower than the average ratings across the three central morphs (−2, 0, +2). While all six patterns are cap-shaped, their average levels nonetheless differ by rater group (adolescents vs. older women, for instance), perhaps as an effect of birth cohort, aging, or sociological context. (We return to this speculation in our Discussion.) Remarkably, the adolescent raters differ substantially from the oldest group of raters in their assessments of the health of the +5 morph as compared to the +2: these groups may have different implicit “theories” of health.

ROW 5, “attractiveness,” *asymmetric cap*. A final pattern is exemplified in the fifth row of the figure, the results for the attractiveness rating. Here, the +5 morph is viewed nearly unanimously as least attractive. While the mean ratings for the extreme morphs deviate from the others in the same direction, those for the +5 morph are more extreme than for the −5. Indeed, there is some agreement here across rating groups that, put bluntly, very heavy faces are unattractive, especially in the minds of male peers. No similar decline is apparent for the exceptionally lean faces at the other end of the morph scale.

## Discussion

The morphometric techniques employed here not only allowed us to isolate, quantify, and plot the facial shape changes that are determined by body fat percentage (BFP), but also enabled the design of facial rating stimuli where only this variable is varied. This experimental control, in turn, allowed us to identify the five distinct response patterns for the attributed traits: flat for the maturity rating, linear sloping upward for the dominance parameter, a combination of these two patterns for masculinity, cap-shaped for health, and a combination of flat-downward with the cap for attractiveness as functions of the morphing parameter BFP. It is this main finding—the dissimilarity of these trend curves down the rating scales together with their similarity across the age-sex groups—that demonstrates the promise of this newly introduced type of stimulus as well as the critical impact of BFP in social stereotyping.

We found little evidence for dependence of a maturity perception on BFP in female adolescent faces. Except possibly for the leanest morph (perceived as being slightly younger), the mean ratings all center around the middle of the bipolar scale, which marks the transition between “child” and “adult”, correctly reflecting the life history stage of the adolescent women used for stimulus production. This finding can also be taken as a quality assessment of the methodology because a fundamental property of the original specimens, their biological age, is correctly recognized in the morph. As with any null finding from a rating study, we cannot entirely rule out rater incapacity or mere carelessness.

For the dominance rating, we found that a more dominant appearance is linear in BFP, with the −5 SD morph being the only one perceived on average as “submissive”. Windhager *et al*.^12^ asked whether the larger, rounded mid- and lower face associated with higher BFP would elicit associations with babies’ chubby cheeks^13,14^ or instead with masculinity and dominance due to the relative jaw prominence^15^. The present finding supports the second of these hypotheses, which is at larger geometric scale. The smaller eyes, the lower eyebrows, and the downturned corners of the mouth in the fatter facial morphs may have contributed to the more dominant and masculine appearance with increasing BFP. The lowest three BFP morphs were rated similarly feminine, whereas the attribution of the +2 SD morph was neutral, and the morph highest in body fat was usually rated as masculine. Scrutinizing the facial shapes involved, the threshold effect corresponds to the facial outline turning from heart-shaped (with a pointed chin) to rectangular (in this case due to an increase of the lower facial width with BFP). Our result is consistent with previous findings that facial cues to body mass index mediate perceptions of strength, which are positively related to impressions both of masculinity and dominance^23,24^.

The cap-shaped response curve for the health ratings offers a functional explanation of the finding from many other studies (e.g.,^25^) that average faces are perceived as healthier than those farther from the average: The finding might reflect the higher health status of average BFP than variants in either direction. Henderson and colleagues^7^ report this same pattern using BMI instead of BFP as the predictor. Coetzee *et al*.^26^ reported a similar curvilinear relationship for the dependence of health rating on a *rating*, not a measurement, of facial adiposity. As there were thus no actual biological measurements in the resulting reported associations, we cannot compare their findings to ours in any numerical way.

Our finding regarding the attractiveness rating can be interpreted along the same lines as the health rating: What is objectively more fit could reasonably be assessed as better. Embracing this logic, Darwinian aesthetics predicts a fitness-dependent relationship between BFP and preference (attractiveness). The vertex of the curve is shifted towards the −2 SD morphs, with the +5 SD BFP rated unanimously least attractive. This asymmetry in the curvilinear relationship between BFP and attractiveness favoring lower BFP faces might be attributable to the relatively good socioeconomic condition and high visual media exposure of the Austrian raters. In developing countries, a low body mass index is a cue to poor health (indicative of diseases inducing wasting) and poverty, whereas in Western industrialized countries, high BMI indicates poor health, while low weight is associated with high socioeconomic status^4,27^. Overall, the curvilinear relationship of BFP with health/attractiveness ratings corresponds well to the biological significance of moderate amounts of body fat storage for female reproductive ability: Both too little and too much body fat compromise many aspects of reproductive endocrinology and reduce fertility^28,29^. Clinical conditions on either end of the body fat scale (such as anorexia nervosa on the one hand and polycystic ovary syndrome on the other) corroborate their disruptive nature.

In summary: BFP is a nonlinear confound in social perception and therefore must be a design covariate in all future studies. These need either to systematically morph for BFP over whatever other dimension(s) of body form are considered for analysis, or else, by explicitly stratified sampling, to control for measured BFP in the collection of facial images.

Across these general patterns, we find some minor rater age-specific sub-patterns consistent with the “other-age effect”^30^. Similarly, there is a decline in face recognition accuracy for young adult faces in older age^31^. One noticeable feature of Figs 2 and 3 is the compression of the range of ratings of the two older rater samples with respect to those of the male and female adolescent raters. This phenomenon appears in each of the five rows of the figures. For instance, the range of the dominance scores for the male peers is the full range of our scale (recoded to 0–100), while the range for the two older groups, especially that for the older women, is narrower. The resolution among the 5 morphs differing only in BFP is apparently higher for the same-age cohort: Our raters seem more perspicacious regarding their peers than older groups. Also, there is a pronounced tendency towards being more discriminating when rating opposite sex peers (who may be construing themselves as potential mates). Keep in mind that it is solely BFP in which these facial stimuli differ. This may reflect the social pressure adolescents experience^32^: Overweight boys and girls are particularly affected by peer teasing and exclusion as well as by parental encouragement to control weight and shape.

Another striking feature is the vertical shift of the curves with increasing rater age towards a more attractive and healthier perception of all five stimuli. Several studies have found age differences in responses to (negative) stimuli, consistent with the hypothesis that older adults show a “positivity bias”^33^.

### Methodological implications

Our study design differs from most of other facial rating studies by virtue of its emphasis on a real biophysical (geometrical) property of the stimulus and its interactions with the two aspects of rater classification, age group and sex. This emphasis strongly shaped our decision to report our findings graphically rather than in the form of tables, *F*-ratios, or *p*-values even when the evaluation of those conventional statistics might be implicit in our data. For instance, while our study’s design substantially diverges from that reported in Zebrowitz *et al*.^33^, the spread of the standard error bars in our Figs 2 and 3 is vaguely analogous to Zebrowitz *et al*.’s correlation-based measure of “agreement”. Equally, the average height (which this paper does not tabulate) of the curves in our figure might correspond to the averaged ratings by rater age and sex in that earlier paper. The focus of our evolutionary hypothesis is the dependence of ratings on calibrated properties of the stimulus. Accordingly, the quantifications of interest in this respect would be not the mean ratings over the set of stimulus images as a whole, but rather the interactions of any of these ratings with the body fatness score that was our main design probe.

### Comments on calibrated stimuli and previous approaches using tails only

The study that originally produced the formula for the shape regression of face on BFP can be referred to as the *calibration study*^12^ as it is completely consistent with the state-of-the-art statistical definition quoted earlier and definition 2 from the Oxford English Dictionary^34^: “Calibrate [transitive verb]. To determine the correct position, value, capacity, etc., of; to set an instrument so that readings taken from it are absolute rather than relative; specifically, to mark (a radio) with indications of the position of various wavelengths or stations.” In this prototypical context, we are marking a “radio” (the deformed average faces) by the positions of various “wavelengths” (BFP scores), since the wavelength is one number, like our BFP, while the radio dial is a location, like any one of our shape coordinates.

We used that shape regression to warp our unwarped average face into the stimuli at BFP −5 SD, −2 SD, 0 SD, 2 SD, and 5 SD from the average BFP as in Fig. 1. Thus each such stimulus has the same pixel values, but at different positions. These regressions are computed by the usual formula, a covariance divided by a variance. The numerator is the covariance of one shape coordinate at a time with BFP; the denominator is a constant, the variance of BFP per se. Importantly, we use regressions over all of the images in the calibration study, not just a comparison of a few faces with high BFP against an equal number with low BFP. This is clearly the best procedure because it yields the most precise regression estimates. This, in turn, provides the most accurately calibrated morphs for later use as stimuli that induce the psychological process whose output (ratings) we are recording. For a formal proof of this assertion, see Appendix 1.

## Conclusion

Thomas Kuhn^35^, who introduced the idea of paradigms in scientific communities, argued that, in the natural sciences, the turn of a discipline to quantification typically comes only after a long prologue during which the community explores the nature of the valid quantifications and the corresponding experimental constraints under which the quantifications are stable. Figures 2 and 3 strongly suggest that the field of evolutionary psychology has not yet arrived at the stage of effective quantification by ratings in that sense. Nonetheless, the method for calibrating stimuli we present here is an important step forward. Moreover, it could benefit a variety of disciplines concerned with social perception in general and with stereotyping as well as stigmatization in particular. For almost half a century, experimental evidence has accumulated that greater attractiveness in schoolchildren as perceived by their teachers leads to higher teacher expectations. The attractive child is deemed more intelligent, more likely to progress in school, and more popular with his/her peers, and its parents as more interested in education^36^. Many other studies show that such stereotypes based solely on appearance persist over the life span of the person being rated, and future research with more fine-tuned measures will no doubt enable deeper insights into such phenomena. Only a design like ours, which forces the human rater to attend to an explicit underlying dimension, can yield findings that permit interpreting that dimension as one cause of the rating behavior.

## Electronic supplementary material

Supplementary Information – Appendix 1 and Appendix 2

Dataset 1

Dataset 2
