# Supplementary material for: Calibrating facial morphs for use as stimuli in biological studies of social perception
Source: Sci Rep. 2018 Apr 27;8:6698. doi: 10.1038/s41598-018-24911-0 (PMC5923288; doi:10.1038/s41598-018-24911-0)
Supplement: Supplementary file 1 — Supplementary Information – Appendix 1 and Appendix 2 [file 41598_2018_24911_MOESM1_ESM.pdf]

Calibrating facial morphs for use as stimuli in biological studies of social perception

Authors:

Sonja Windhager[1,*], Fred L. Bookstein[2,3], Hanna Mueller[2], Elke Zunner[2], Sylvia Kirchengast[2], Katrin Schaefer[2]

Supplementary Material

Appendix 1

Appendix 2

Appendix 1. Demonstration that the precision of a regression is highest when a sample is not truncated to the extremes

It is easiest to show the superiority of regression on the full sample over regression on any subsample if we assume an optimally designed study for which the distribution of BFP is actually *uniform* over the calibration sample. In this situation, the argument is as follows. The formula for the sampling variance of a regression coefficient—the quantity we want to make as small as possible—is

$\mathrm{var}(\beta) = \mathrm{var}(\varepsilon)/[n\ \mathrm{var}(x)]$ where β is the regression coefficient, ε is the true error in the model, x is the predictor, and n is the sample size involved in such a calibration study. If the distribution of x is uniform over some interval—e.g., from −1 to +1—then the var(β) given by this formula continuously rises [which we DO NOT want] as the thresholds $-k$ and $+k$ at which we decide to censor our sample move away from zero to the extremes of ±1. In other words, we are considering restricting the predictor to the pair of intervals $(-1, -k)$ and $(k,1)$, and we will show that var(β) rises as a function of k. We start at k=0, the two "50% tails" that, when combined, preserve our whole sample. For that full distribution of predictors x uniformly distributed over the interval from −1 to 1, the predictor variance is only 1/3.

For predictors that are uniformly distributed: Those two intervals, combined, represent a fraction $(1-k)$ of our original sample count. The variance of x is the mean of x^2 over the interval from k to 1, which is the integral of x^2 over that interval divided by the length of that interval. As $\int x^2\, dx = x^3/3$, the integral is $(1-k^3)/3$, divided by $(1-k)$. But the quantity n in the formula for var(β) drops precisely in proportion to $(1-k)$. The two factors of $(1-k)$ cancel each other, leaving, in the formula for the denominator of var(β), the integral we started with, which was $(1-k^3)/3$. This is clearly a maximum, and hence the variance of β a minimum, when k=0—when our two "tails" together use all the data. That value is 1/3, the value we already had. k^3 rises to half its maximum, and hence $(1-k^3)$ falls to half of *its* maximum, for k^3=0.5, k=0.794—about the setting of 10% and 90% tails. There **the variance of the estimated regression slope is merely double the optimal precision**. Ironically, one often finds exactly this tail threshold in papers of our tradition.

For other predictor distributions, such as the Gaussian: A similar argument can be mounted for predictors x that are distributed in distributions other than the uniform. For a Gaussian predictor, for example, the denominator of the standard formula will be larger than

the denominator of a 10% – versus – 90% tail comparison by the product of 0.20 (the fraction of your sample you are using) times the mean square of that Gaussian tail around zero. The tail specification cuts the Gaussian at about 1.28 standard deviations; the mean square of the bell curve tail above that is about 3.23. 20% of 3.23 is about 65%, making the efficiency of estimating a regression slope from the extreme 20% of a Gaussian sample of predictors about 65% of what it would be by simply regressing on everybody. For a 5%–95% tail comparison, the Gaussian threshold is 1.645 standard deviations, the predictor variance is 4.4, and so the corresponding efficiency is 10% of that, or 44%, which is even worse than the 50% ratio of the 10–90 tails of the uniform predictor. In short, the strategy of tail-comparison is never the best way to produce a continuously morphing stimulus for studies exploiting the psychophysical design that we are recommending here for investigations into psychomorphospace.

Appendix 2. Comments on statistical significance testing

Colleagues who reviewed early drafts of this manuscript noted the absence of conventional statistical significance tests. Indeed the specific design of this study does not conduce to any conventional testing scheme.

Consider the following three crucial design features:

(1) repeated measures over traits—each subject rated all five of the traits we are reporting;

(2) repeated measures over stimuli—each subject rated all five of the stimulus levels (the settings of body fat percentage at −5, −2, 0, +2, or +5 standard deviations away from the mean);

(3) the discretization of those stimulus levels, along with the intentional exaggeration of the natural range (real data do not often approach levels of ± 5 SD).

In a study this complex, typical notions of analysis of variance and covariance do not apply. The definition of a "sum of squares for interaction," for instance, does not work when a continuous variable appears in an analysis by way of two different polynomial terms (linear and quadratic) that have not been orthogonalized (so that we could consider the effects of exaggeration explicitly). "Masculinity" and "dominance" are surely correlated as ratings, but there is no conventional language of reporting such correlations over the doubly-repeated aspects of the present design.

For all these reasons, we concluded that no finding of a conventionally "significant" effect, according to any of the available general linear models and their output tables, could be properly reported. Were we to ignore the doubly-repeated design here, and also the intercorrelations among the ratings of the several traits, the only valid reports would be those saying that each of the trait-by-trait analyses is "significant." But inasmuch as there *are* correlations among the ratings, both by rater and by stimulus, even that language is misleading. The only possible valid report of a "significance level" would be the report of a p-value for our study as a whole. We suggest that such a report be superseded by the simple fact of publication, along with the patterns that are so plain in Figures 2 and 3.

There is no doubt that our study has arrived at scientifically meaningful contrasts. The conventional language of variance decomposition would decompose our findings into separate "effects" of age and sex, linear and quadratic trends over the stimuli, and all the two-way and three-way interactions among these factors. We do not believe any such decomposition is justified in terms of the actual process(es) we are observing, and therefore we do not report any significance levels at all. In short, we maintain that the scatterplots in Figures 2 and 3 speak for themselves, without the need of any formulaic arithmetic intervening.
